# A qualitative study of community pharmacists in New Zealand: mental health literacy and the barriers and facilitators to providing and receiving mental healthcare in community pharmacies

**DOI:** 10.1016/j.rcsop.2025.100621

**Published:** 2025-06-08

**Authors:** Frederick Sundram, Amy Hai Yan Chan, Joanne C. Lin, Retina Rimal, Timothy F. Chen, Jane L. Sheridan

**Affiliations:** a Department of Psychological Medicine, Faculty of Medical and Health Sciences, The University of Auckland, New Zealand; b Centre for Brain Research, Faculty of Medical and Health Sciences, The University of Auckland, Auckland, New Zealand; c School of Pharmacy, Faculty of Medical and Health Sciences, The University of Auckland, New Zealand; d School of Pharmacy, Faculty of Medicine and Health, The University of Sydney, Australia; e Centre for Addiction Research, Faculty of Medical and Health Sciences, The University of Auckland, Auckland, New Zealand; f School of Population Health, Faculty of Medical and Health Sciences, The University of Auckland, Auckland, New Zealand

**Keywords:** Mental health literacy, Community pharmacists, Mental health, Stigma, Health literacy, Attitudes, Service delivery, Primary care

## Abstract

**Background:**

Community pharmacists (CPs) play a valuable role in the provision of mental healthcare in primary care settings; however, CPs may not be comfortable or confident doing so. Mental health literacy (MHL) of CPs and consumers, alongside factors in community pharmacies may contribute to this.

**Objective:**

The aims of this study were to explore how MHL may affect the provision and receipt of mental healthcare in community pharmacies, and the perceived barriers and facilitators for CPs in providing mental healthcare.

**Methods:**

Participants were recruited from a sample of respondents to a prior national survey of MHL in CPs. Thirteen CPs with a mean age of 40 years, comprising participants from both independent and chain pharmacies were recruited. Individual, semi-structured interviews were conducted, followed by professional transcription and thematic analysis.

**Results:**

Analysis revealed five main themes describing factors related to MHL and also the provision of mental healthcare services in community pharmacies: 1) Complexities in the understanding of and recognition and management of mental illness; 2) Attitudes and experiences of CPs in supporting mental health needs; 3) Prevention and management of mental illness needing a collaborative approach; 4) Opportunities and challenges within community pharmacies to support mental healthcare needs; and 5) Preparedness and willingness of CPs to provide mental healthcare.

**Conclusions:**

CPs identified several MHL-related factors that could affect mental healthcare delivery by CPs and consumers receiving care. CPs also described several ways to enhance preparedness to deliver mental healthcare in community pharmacies including working collaboratively with other healthcare providers.

## 1 Introduction

Mental health and addiction problems are increasing worldwide with the cost of mental illness estimated to be US$16.3 trillion between 2011 and 2030 to the global economy.^1^^,^^2^ This is associated with severe impacts on standards of living and socioeconomic development.^3^ Each year in New Zealand (NZ), one in five New Zealanders experiences mental illness and approximately 80 % experience distress.^4^ Further, around 7.8 % of the population in NZ reported unmet need for professional help for their emotions, stress, mental health and addiction in 2022, which is almost double that in 2017 (4.9 %).^5^ As part of a stepped-care approach in addressing the spectrum of mental health needs from mild through to severe, primary healthcare teams can play a critical role in the identification and management of mental illness, and provision of support to individuals with mental health needs.^6^ The integration of community pharmacists (CPs) as a part of such an approach is encouraged, but strategies to better integrate CPs need further development in many countries^7^ including in NZ^8^ despite CPs being well-positioned to support mental healthcare in primary care settings.^9^

CPs are the most accessible and trusted healthcare providers to the public and often serve as an initial point of contact for people living with physical or mental health needs.^10^^,^^11^ Indeed, the accessibility of CPs was evident during the COVID-19 pandemic, as pharmacies remained open and provided essential services and access to healthcare. This was particularly important in rural and regional areas of NZ, where timely access of healthcare services is limited. Additionally, CPs are recognised as medicines experts and an important profession for supporting mental healthcare and psychological wellbeing^12^ and medication adherence^13^ as medications can play an essential role in the treatment of many mental health conditions. In 2018, around 425,000 New Zealanders received psychotropic medication regularly^14^ while an Australian study showed increased psychotropic medication prescribing in general practices during the pandemic.^15^ CPs can also support improvement in adherence and reduction of prescription of inappropriate psychotropics in mental illness and addictions.^16^^,^^17^

Moreover, the role of CPs in supporting mental health can include screening and recognition of mental illness; providing psychoeducation; advocacy and; signposting of and referral to other services.^7^ A recent systematic review of pharmacy-led interventions for individuals with persistent mental illness found a positive impact on adherence and quality of life and a reduction of disease progression and hospitalisations.^18^ Depression screening programmes led by CPs have also been shown to increase the early detection of depression, leading to interventions in people who may otherwise have not sought help.^17^ Similarly, pharmacist-led educational interventions for mental health have been shown to improve medication adherence, levels of knowledge and quality of life.^18^^,^^19^ Further, with regard to subthreshold depression and anxiety associated with long-term physical health conditions, CPs have a role in preventing the progression of these conditions across the clinical threshold.^20^ In that study, interventions offered by CPs that were found to be potentially useful included problem-solving therapy and behavioural activation. This suggests that CPs have a role to support mental healthcare in a holistic way regardless of whether there is a primary mental illness or secondary to physical ill health.

International pharmacy bodies have advocated for the role of pharmacists in mental healthcare, including the Pharmaceutical Society of Australia, the Royal Pharmaceutical Society in the UK and the International Pharmaceutical Federation highlighting the importance of pharmacists in this clinical area.^21^, 22., 23., 24 However, pharmacists need to have the requisite knowledge, skills and attitudes in order to be able to deliver effective mental healthcare; internationally, studies on the training needs of pharmacists have found that developing competencies around communication skills, managing crisis presentations and knowledge around mental health, psychotropics and addressing stigma are required.^25^, ^26^, ^27^, ^28^, ^29^ Importantly, underlying many of these various key factors is adequate mental health literacy (MHL).^30^ MHL is defined as “knowledge and beliefs about mental disorders which aid their recognition, management or prevention.”^31^ MHL is a multifaceted concept which has been further refined since it was originally described and now encompasses several components: “(a) the ability to recognise specific disorders or different types of psychological distress; (b) knowledge and beliefs about risk factors and causes; (c) knowledge and beliefs about self-help interventions; (d) knowledge and beliefs about professional help available; (e) attitudes which facilitate recognition and appropriate help-seeking; and (f) knowledge of how to seek mental health information.”^32^

Studies among CPs and pharmacy students have identified a poor understanding of mental illness.^33^, ^34^, ^35^, ^36^ Additionally, studies of the MHL of CPs in Australia and UK found that while CPs were able to recognise depressive symptoms, they were less comfortable with providing services to those with depression, bipolar disorder and schizophrenia compared to cardiovascular disease.^30^^,^^37^^,^^38^ Studies from the United States^39^ and Canada^40^ have similarly found limited levels of confidence and comfort in discussing mental health symptoms or psychotropic medications with consumers with a mental health diagnosis despite willingness, positive attitudes and interest in CPs to support mental health. This may suggest an interplay of MHL with other factors in the community pharmacy setting that may affect confidence and comfort during interactions with those with mental health needs. These factors may include time available for consultations, the business philosophy of the pharmacy, the workplace environment and degree of collaboration with other healthcare professionals.^7^^,^^8^^,^^41^

Though MHL is an important factor for non-mental healthcare professionals in recognising mental illness and providing support,^42^ there is currently a lack of research on the MHL of CPs and other factors that may affect mental healthcare delivery in community pharmacy settings in NZ. There are notable differences between NZ and the UK and Australia where much of the prior research has been conducted among CPs. For example, the density of pharmacists is lower in NZ compared to other countries^43^ and NZ has a large indigenous (Māori) population with unique cultural beliefs and practices that influence perceptions and approaches to mental health.^44^ In terms of community pharmacies, there are approximately 1100 such pharmacies in NZ that can be categorised as the following: 1) independent or owner-operated 2) chain 3) supermarket brand and 4) corporate or discount.^45^ In 2022, there was a reorganization of the healthcare system with the amalgamation of district health boards across the country that covered various regions into a unified healthcare entity (Te Whatu Ora/Health New Zealand) with the aim of providing more consistent care across health districts and overcoming fragmentation across services. Community pharmacies that had started moving away from a predominantly transactional approach prior to this reorganization, continued to do so, with more focus on health service provision.^45^

Studies of mental healthcare in community pharmacies in the NZ-context are limited or have been undertaken a while ago.^46^^,^^47^ In previous studies, CPs were found to play an important role beyond the provision of medication or medication advice and contribute in a variety of ways including building relationships, providing education, psychological support and initiating referrals.^8^^,^^46^^,^^47^ A recent national online survey examining the MHL of CPs in NZ found a high degree of MHL and positive attitudes towards mental health, but CPs lacked confidence and comfort in delivering aspects of mental healthcare.^48^ Moreover, CPs wished to gain training in specific aspects including pathways for referral to specialist mental health services and also support to communicate better with those with mental health needs. That study was important in identifying key aspects of MHL and needs of CPs. This study builds on the findings of the prior MHL survey by using qualitative interviews to explore: 1) how MHL may affect the provision and receipt of mental healthcare in community pharmacies, and 2) the experiences and, perceived barriers and facilitators for CPs in providing primary mental healthcare in a community pharmacy setting.

## 2 Methods

### 2.1 Study design

Following on from the findings of a national survey of MHL in CPs,^48^ this study used a qualitative design incorporating a semi-structured interview method which allowed for interviews exploring individual perspectives in-depth. Ethics approval was obtained from the University of Auckland Human Participants Ethics Committee (UAHPEC Reference 023957).

### 2.2 Participants and recruitment

Participants were recruited from the sample of respondents who participated in a prior survey of MHL in CPs.^48^ While the participants in the current study are a sub-sample of the larger quantitative study, participant responses were decoupled from their demographic data as per our ethics approval requirements. After completing the prior quantitative survey, the participants completed a separate delinked survey whereby they only provided their contact details (email and phone number) if they were willing to participate in a future qualitative study. Of the 346 participants who completed the online quantitative survey, 85 participants subsequently completed the delinked survey and were thus potential future participants who could be approached for the current qualitative study.

The 85 participants who provided their details after survey completion were emailed an invitation to participate in this study. A reminder email was sent, followed by a text message to recruit participants if there was a non-response to initial contact. To recruit a range of participants, we utilised a sampling frame whereby potential participants were asked to provide details on their years of pharmacy experience (less than 10 years vs. 10 years or more) and type of pharmacy they worked at (independent vs. chain pharmacy). Participants were provided with an information sheet and signed consent was obtained prior to the interviews. They received an NZD$50 shopping voucher as a token of appreciation and to acknowledge their time for participating in the study.

### 2.3 Data collection

A semi-structured interview guide was drafted based on prior related literature^17^^,^^30^^,^^31^^,^^49^ and the results of the national MHL survey in NZ.^48^ Two pilot interviews to test the interview guide were undertaken with two senior academic pharmacists with previous experience in community pharmacy, recruited via professional networks. They were provided with background information regarding the purpose of the study. Feedback was collected from the pilot interviews where questions were reviewed for clarity, ease of understanding and relevance and interview questions were further refined to develop the final interview guide. The main interviews were conducted online individually via the Zoom video conference platform and audio digitally recorded, between March and September 2021.

### 2.4 Data analysis

The recorded interviews were transcribed verbatim by a professional transcriber. The transcripts were verified for accuracy by reading transcripts and listening to recordings concurrently by one researcher (RR). Participants were offered the chance to review their transcript. NVivo® (Version 12.0) (NVivo qualitative data analysis software; QSR International Pty Ltd.) was used to organise the data. Transcripts were analysed using thematic analysis.^50^ Three authors (RR, FS, AC) coded the first two transcripts independently. The codes were then discussed with the rest of the authors, and any disagreements resolved by consensus discussion to develop an initial coding framework. The coding framework was then used to code the remaining interviews. After all the transcripts were coded, two members (RR and FS) of the team discussed the coding and identified the main themes. The themes and their associated codes were shared with the entire team. The themes were reviewed by members of the team (RR, FS, AC, JL, JS) and were finalised along with the sub-themes. Data saturation was determined when no new codes were found in the data.

## 3 Results

The first invitation email elicited a response from 9 CPs while an additional four participants were recruited via a reminder email. Recruitment ended after 13 CPs were interviewed when data saturation had been reached. Interviews lasted between 25 and 60 min and Table 1 summarises the characteristics of the participants.

**Table 1:** Characteristics of included participants.

Characteristics	Summary
	n (%)
Age (Mean, SD)	40 years, 14.3
**Gender**	
Female	9 (69 %)
Male	4 (31 %)
**Ethnicity**	
NZ European	7 (54 %)
Chinese	3 (23 %)
Others (Fijian Indian, Indian, Malaysian)	3 (23 %)
**Current position**	
Manager	2 (15 %)
Locum	3 (23 %)
Pharmacist	6 (46 %)
Owner	1 (8 %)
Intern	1 (8 %)
**Length of experience as a pharmacist**	
Less than 10 years	8 (62 %)
10 years and more	5 (38 %)
**Type of pharmacy**	
Chain	4 (31 %)
Independent	9 (69 %)

### 3.1 Participants

Participants had a mean age of 40 years, with two thirds having less than 10 years' experience in practice. The majority of participants worked in independent pharmacies, were female, and of NZ European self-identified ethnicity (see Table 1).

### 3.2 Themes

Analysis of the interviews generated five main themes in relation to MHL and the provision of mental healthcare services in community pharmacies: 1) Complexities in the understanding of and recognition and management of mental illness; 2) Attitudes and experiences of CPs in supporting mental health needs; 3) Prevention and management of mental illness needing a collaborative approach; 4) Opportunities and challenges within community pharmacies to support mental healthcare needs; and 5) Preparedness and willingness of CPs to provide mental healthcare.

#### 3.2.1 Complexities in the understanding of and recognition and management of mental illness

Participants reflected that mental illnesses are often complex, long-term conditions that encompass a range of disorders, each requiring appropriate communication to explore symptoms further.

“*Mental illness encompasses a range of different symptoms and a range of different severities and it impacts a lot of people in a lot of different ways.”*

*- Participant 11*

Participants considered mental illness as different from physical illness due to the lack of physical manifestations in most mental illnesses which can lead to a late diagnosis and delays in treatment. Participants also shared that the subjective experience of mental health necessitates a more personalised approach and a longer time-commitment when mental healthcare is provided.

*“it may be more so about thought processes versus something like a fracture where you can physically see the bone being broken. It's more so individual's experience rather than having external symptoms that maybe like a scan could pick up.”*

*– Participant 6*

*“mental health conditions is (sic) messy, you've got to actually go in there and actually talk to the person and explain it and be involved with a person and on top of that it's going to take longer.”*

*– Participant 12*

Participants noted that, unlike physical health issues, consumers hesitate to seek help for mental health issues. Participants shared that the understanding of mental health is often shaped by social norms and cultural beliefs. They expressed concerns about societal pressure to appear “normal,” and how mental health challenges are often viewed as being outside the norm and stigmatised. Participants discussed how their own understanding of mental health is influenced by their own cultural beliefs.

“*mental illness is sort of inward feeling with weakness and shame associated with it, so, I think it also sort of breeds secrecy not telling other people and not seeking help”*

*– Participant 13*

*“in our culture mental health conditions is seen as like something that you don't really talk about, you don't really discuss, it's something that's almost like a shame to the family or like shame to the people around you. So there definitely is cultural difference.”*

*– Participant 6*

Participants also noted the impact of media depictions of mental illness, which could lead to stigma among CPs and wider society, and the potential role of the media and government in addressing this with public health and educational campaigns. Furthermore, participants suggested the role of society in destigmatising mental illness by discussing, sharing and caring more about these issues.

*“I think in the media I would say that movies are probably the worst thing for mental health because they take like a schizophrenic and so forth and they make them a mass murderer, so everyone thinks that all schizophrenics are mass murderers. I think that in this day and age it's really hard to rely on one media, the reason being is that people nowadays, back in the, what is it, fifties, sixties, seventies, a large portion of people got most of the news from the newspaper or from the TV right.”*

*– Participant 12*

*“I think the media doesn't portray enough of mental illness, like whenever it does it always be like someone did a bad thing so that's why it's on the news.”*

*– Participant 7*

*“I guess there is a lot more education and currently in the media there's a lot more discussion and a lot more normalisation of mental health issues. I know there's been some celebrities that have done, talked about their feelings of depression or talked about their struggles with mental illness on social media and in the newspaper and things like that.”*

*– Participant 3*

*“I think as a whole cultural shift, that needs to come from the media or the Government. So I think, yeah, community pharmacists you can do really, a lot of great, good work for individuals but it's very difficult to impact on a community basis.”*

*– Participant 9*

#### 3.2.2 Attitudes and experiences of CPs in supporting mental health needs

Participants expressed uncertainty, discomfort and a lack of confidence during interactions with someone with mental illness which could lead to presumptions and a reluctance to engage among CPs. This may be related to unfamiliarity of how to communicate with someone with a mental illness without offending them and how to provide appropriate supports when someone was unwell.

*“Some CPs are concerned the person with mental illness may start crying or get angry when you approach them”*

*– Participant 3*

*“the general discomfort or unfamiliarity around dealing with mental illness creates reluctance in CPs”*

*– Participant 8*

There was a perception that people with mental illnesses may not be able to take onboard or understand information and, therefore, it could be challenging to have discussions though CPs recognised the importance of not treating consumers with mental illness differently.

*“I would not feel confident approaching somebody that was experiencing some kind of delusion. I would sort of worry about what's the right thing to say and what's the right course of action to help this person”*

*– Participant 3*

*“I won't rely so much on the diagnosis but I think the counselling skills, even if it's like, even if somebody, you notice that one of your normal patients is feeling a bit down and stuff, like some tips on how to start the conversation around mental health, how to ask them if everything's okay in terms of how they're feeling generally…”*

*– Participant 5*

Participants emphasised that the most crucial attributes for CPs to be able to help people with mental health needs were personal resilience, compassion, and empathy. Participants believed that these attributes developed with professional experience, exposure to mental illness and personal experience of mental health challenges. Possessing these qualities helped CPs feel more comfortable and confident in approaching individuals with mental health needs.

*“empathy but yet resiliency in dealing with people who are struggling, they both go hand in hand, rather than reacting to them, you actually have this true form of empathy where you see them for where they are and where they have come from”*

*- Participant 1*

*“I feel like being around people with mental illness have made me more empathetic towards people just because I know how hard it is”*

*– Participant 11*

*“the personal experience I had made me see there was a person in need and that I could actually do something as a community pharmacist”*

*– Participant 2*

Participants raised the issue of stigma in terms of barriers to both mental health related help-seeking by consumers and help providing by CPs. Some participants discussed the presence of stigmatising beliefs among CPs, leading to prejudice that affects their colleagues' clinical practice. Participants suggested that workshops or training from individuals with lived experience of mental health challenges could help CPs develop the necessary knowledge, skills and attitudes alongside a compassionate approach to support people with mental health needs. Recommendations also included shadowing other pharmacists with professional experience in mental health.

*“hear from people that are living with these illnesses that I'm not so familiar with, to understand their experience and how they feel we could help them best as health professionals, to understand what they would want from us and how best to deliver that but also just to understand what they're living with”*

*– Participant 3*

*“working closely with pharmacists who have extensive experience in a mental health ward, would be extremely helpful”*

*– Participant 11*

Several participants recognised the importance of self-care to maintain their mental wellbeing and how it could be applied to both them and to the consumers they encounter.

*“I exercise regularly. I practice sleep hygiene. In terms of going off, this is another thing I tell people, especially teenagers if they're having treatment for illnesses is to go off social media.…. I'll practice sort of things like breathing exercises or say going to a garden centre or whatever. Yeah, make time to catch up with friends, prioritise, socialise and things like that”*

*– Participant 8*

#### 3.2.3 Prevention and management of mental illness needing a collaborative approach

Though participants recognised that they, as CPs, are the most accessible healthcare professional; however, they mentioned the need to involve various healthcare professionals including psychiatrists, psychologists, general practitioners (GPs) and CPs as part of a team.

*“we are the health professionals they see most often, and we are most accessible. They can come to us without an appointment and without having to pay a fee for consultation”*

*– Participant 5*

*“it would be quite nice to be part of a team providing a response, we can possibly reinforce the messages from other health professionals”*

*– Participant 3*

With the configuration of the NZ healthcare system currently, GPs were identified as the first point of contact and participants thought that GPs and other healthcare professionals were responsible for the overall management of mental illnesses, with CPs in a supporting role. Participants noted that CPs can provide information on medications, reinforce treatment plans and support the recovery and empowerment of consumers, thereby having an important role in the prevention of mental illness and the promotion and maintenance of mental health of consumers and their families.

*“GP initially, and they could further direct them to either a therapist, psychiatrist, or psychologist”*

*– Participant 4*

*“It's more about aiding in executing the management plan formed by the doctor. So, it's to reinforce and support the management of that patient”*

*– Participant 9*

*“empowering someone to do the best they can for themselves at home helps recovery best. I think pharmacists are in a really good place to encourage people to take care of themselves.”*

*– Participant 8*

*“we're still the health professional people see most often and by helping people empower themselves to take care of their own health, that also helps them take care of others”*

*– Participant 8*

Participants also talked about a medication monitoring role for CPs, having the ability to see consumers frequently and shared examples of collaborative approaches in their community with other healthcare professionals including times when someone might be becoming unwell or relapsing with mental illness and seeking earlier appointments with the main treatment provider.

*“I think monitoring of medication is something we can do as we have the ability to check their records and see them frequently”*

*– Participant 4*

*“We can pick up on red flags or missed doses of medications and then report it to the doctor who manages those observations.”*

*– Participant 13*

*“I know all of the doctors, all the mental health workers, all of the staff there and I'm able to just pick up the phone and say I've got so and so here, he's really struggling, is there any possibility we could get an appointment and nine times out of ten they'll accommodate”*

*– Participant 12*

One of the reasons for better collaborative relationships that have been established was proximity to other healthcare disciplines such as being located within a primary healthcare building. Participants also suggested ways to improve collaboration such as regular meetings with other healthcare professionals, developing a working relationship with GPs and other healthcare professionals in the community and a shared electronic patient platform.

*“I think collaboration would be a good idea actually to introduce to the, introduce in the primary care, like collaboration between the GP and the pharmacist where we have patients like this, selected patients maybe who need extra care, maybe if we have monthly or weekly meetings to see how they're going, if they're picking up their repeats, how we think they are, how the GP can give, that will be really good”*

*– Participant 5*

#### 3.2.4 Opportunities and challenges within community pharmacies to support mental healthcare needs

Though participants would like to provide more mental healthcare within community pharmacies, most participants recognised that the challenges for CPs in providing optimal mental healthcare were a lack of resources and time. Participants noted that capacity in terms of staffing and financial pressures have led to constraints in the amount of time spent with each consumer, and the pressure on CPs and resulting stress can eventually lead to a depersonalised service. Participants also identified an increasing number of locums working particularly in chain pharmacies which participants believed could further create a depersonalised model in community pharmacies for consumers. While these factors may affect the provision of health-related services in community pharmacies in general, these may particularly impact the delivery and receipt of mental healthcare in community pharmacies given the need for building relationships and the time required to explore symptoms.

*“the pharmacy I work at is a really, really busy pharmacy and its big, that's actually a workload of like over four hundred scripts a day, there is just no time to talk to people”*

*– Participant 8*

*“there are locums working every day, so when you go to pick up your medicine sometimes you see this pharmacist, sometimes you see that pharmacist and basically I feel it's becoming more of a corporate business than health service offering”*

*– Participant 5*

*“I think if places [pharmacies] had better resources and were staffed more appropriately, that would be a big factor that would improve things in mental health space”*

*– Participant 3*

Additionally, system barriers such as a lack of scope for communication between healthcare professionals, a fragmented healthcare system, unclear referral pathways and long patient waitlists to access specialist mental healthcare were identified as potential barriers to the mental healthcare services CPs could provide. To overcome system level barriers, participants recommended a shared electronic patient platform to promote collaboration between healthcare professionals and potentially overcome fragmentation within the healthcare system. Participants also identified the need to have appropriate workforce staffing levels and improved funding by government to improve the breadth of services in community pharmacies and to create an overall responsive specialist mental healthcare system for those seeking help that primary care is unable to manage. Additionally, most participants did not have a pharmacy dispensing checking technician available in their pharmacies - these are roles that would potentially allow pharmacists to spend less time on medication dispensing and more time on clinical interactions with consumers attending their pharmacies.

*“a shared platform so I can actually put in new information into that patient's shared medical record, that's then received by the health provider at the other end and that's how the system should work”*

*– Participant 1*

*“money is time and time is money and so if you are able to free up the pharmacist by having good technicians, good technicians are worth their weight in gold, they then can do all the work behind the scenes”*

*– Participant 12*

*“Usually there is no scope for discussion with the GP or even Mental Health Services because we are only at the receiving end of the prescription. So, whatever happens in their lives we are mostly not aware of because we save the script, check if the dosage is right and give it out”*

*– Participant 7*

*“I mean we're a multidisciplinary healthcare team so we should be actively involving with each other to discuss patients and that is what I meant by a pharmacist's role is to pick up on these red flags or pick up on missed doses of medications and then report it to the doctor who then manages those observations”*

*– Participant 9*

#### 3.2.5 Preparedness and willingness of CPs to provide mental healthcare

As knowledge about mental health is a key aspect of MHL and influences the development of mental health-related skills, when and how participants had gained their knowledge was explored. All participants noted that it was from required learning that was part of their undergraduate pharmacy curriculum at university, but participants expressed concerns about not having enough knowledge and skills to deliver care to consumers with mental health needs. However, if CPs developed a particular interest in mental health, participants noted that they engaged in upskilling by reading and going to training sessions.

*“ever since Uni, we did more practices on things like cardiovascular disease, cholesterol, glucose, all that stuff because there are a lot of published studies that show that hey this diet works, this lifestyle factor works, this medication was better than the other and what's the best time of day to take it and all that stuff but for topics around mental illness, our University usually puts them at the last year or as close to the last year as possible because there's so much uncertainty and one type of illness may have symptoms that overlap with another type of illness and there are so many things that we can just assume about people”*

*– Participant 7*

*“I went on training on the side effects of common mental health medications and how to support patients through them because there's a lot of nasty side effects that, on really important medications and so its balancing that risk benefit profile and how do you have those discussions with patients so that they're both making an informed decision”*

*– Participant 1*

Most participants showed enthusiasm to provide mental healthcare in their community pharmacies and wanted to work at the top of their scope of practice which would include clinical interactions rather than being in a predominantly medication dispensing role. Some pharmacies had consulting rooms which would allow these clinical interactions to occur in a private space. Participants recognised their limits in terms of scope of practice and the type of mental health needs they could manage before referring on to other appropriate healthcare professionals. However, a few participants indicated that they were considering leaving the profession as they were unhappy with the way community pharmacy services were currently configured, they had mental health needs of their own and felt underappreciated.

*“best way maybe pharmacists can help is definitely with establishing that relationship with their patients and kind of just making it seem like it's okay like kind of destigmatising so just make it feel like even if someone did have mental health issues that it's not a big deal or that it's common just by having posters or pamphlets or those types of things”*

*– Participant 11*

*“Like we have consultation rooms, we can talk about things privately, we can see people on a more regular basis, like if they want to book in and make an appointment however long, like every week or every two weeks initially and then see how we go then we can just educate them on what kind of treatments might be available or if we can provide maybe a more scientific perspective of mental illness, I think that would be quite good.”*

*– Participant 6*

*“I can certainly listen, give advice, tell them what I think is best but if they're suicidal, I'd probably say that I'm very grateful they've trusted in me for help but I think it's best if we get involved a doctor of some sorts”*

*- Participant 9*

*“I personally am seeking to leave pharmacy as a profession because I just feel like it's not the right fit for me at the moment, so it's, it's hard for me to say without being biased but I definitely think that the Pharmacy Council or whoever can definitely be doing a lot more to help pharmacists with their, just seeing that their mental health needs are taken care of or to see that they're even being appreciated”*

*– Participant 11*

Participants suggested various topics of training that could be useful to improve their MHL including recognising symptoms of a variety of mental health conditions and how to pick up on major concerns which were considered red flags.

*“think it would include things to look out for in patients. So, what they call red flags, so that you would be more aware of when to refer because obviously as a pharmacist you're the first point of call for most people, so you can often see patterns over time in people's behaviour and health. So, a huge thing of what I would like to see in training would be red flag referrals and actually hopefully how to talk to people with mental health issues and how to normalise it I think would be a really nice or good training to have”*

*– Participant 9*

Additionally, improving communication skills so that they could build rapport with consumers and support better with discussions for example, around mindfulness and self-care rather than referring on to other healthcare professionals being the main option for management.

*“so I definitely think that it is more specialised like the communication, the tools, the tactics, the words that you use, the questions you ask whether they're open ended or closed questions, like it would definitely vary between a person with mental health issues and a person without and we can't do that unless we are taught how to do those things”*

*– Participant 11*

*“counselling or mindfulness because perhaps that would be a good starting point for some patients instead of us saying, ‘Hey have you considered talking to a doctor,’ you know, like it depends on their comfort level or if they want to maybe self-manage it for a little while, see if they can do it before escalating it if need be”*

*– Participant 6*

Some pharmacists noted that their professional experience and exposure to mental illness, upskilling and involvement in self-care had built confidence in dealing with mental health needs of consumers. Some participants had engaged in specific “on the job” training through their professional society, for example on clozapine use, and workshops on anxiety disorders and depression. However, participants suggested several areas that they would like further experience and training in.

*“I do see them often and I think confidence comes with experience. I have been in practice for quite a long time you know.”*

*– Participant 4*

*“I feel like it's made me more empathetic towards people just because I know how hard it is personally and with people that have come in, ….. we might say like it's really hard because you're like you need to talk to these things about like with someone”*

*– Participant 11*

*“I think it should be multifaceted and very specific so that you can focus in on various aspects of mental health, like it might be anxiety, it might be different types of anxiety, it might be different types of depression, it might be different types of schizophrenia, it might be coping mechanisms, it might be tools to deal with various aspects of life changing experiences and pull together from the resources”*

*– Participant 12*

## 4 Discussion

This study explored how MHL can affect the provision and receipt of mental healthcare by CPs and consumers respectively as well as the experiences, barriers, and facilitators for CPs in providing mental healthcare in community pharmacies. Based on interviews of 13 CPs with varied professional experience and place of practice, our findings demonstrate that CPs believe they play an important role as a frontline provider in supporting people with mental health needs.

### 4.1 Barriers and facilitators in providing effective mental healthcare

Most participants were willing to provide mental healthcare in community pharmacies and recognised the value in providing mental healthcare services that were personalised. The CPs identified positive attitudes, professional and personal exposure to mental illness, upskilling, the presence of pharmacy dispensing checking technicians and collaboration with other healthcare providers as some of the facilitators to providing optimal mental healthcare in community pharmacies. However, the participants identified mental illness as a complex issue, and this complexity could contribute to discomfort for CPs as they may not always have the knowledge or skills to recognise mental health disorders, engage with or provide ongoing support to people with mental health needs. Additionally, all participating CPs reported characteristics such as stigma around mental illness as a barrier for both help-seeking among consumers and help provision by CPs. Moreover, several pharmacy-related factors (lack of time and resources, increase in chain pharmacies, high prevalence of locums); and system-related factors (fragmented healthcare system, lack of collaboration between health professionals) were identified as barriers. A study undertaken in Australia suggested a framework that includes better remuneration and recognition of CPs' competencies as well as improved integration, standardised practice and public awareness would be required to enable better mental health support in community pharmacies.^51^

### 4.2 Roles and responsibilities of community pharmacists in mental healthcare

Most CPs in this study perceived their responsibilities in mental healthcare as broad and included recognising symptoms, making referrals to other healthcare professionals, and managing and supporting psychotropic medication-related problems, which aligns with the existing literature internationally.^16^^,^^52^ Additionally, participants described the accessibility of community pharmacies and CPs as medicines experts who can provide patient support in the form of optimising psychotropic medication adherence and raising red flags for example when a patient stops medications, consistent with recent reports on the roles of CPs.^53^ Participants supported CPs potentially initiating referrals to other services for those who have mental healthcare needs suggesting knowledge of the appropriate professional supports available and the wish to provide an integrated response to mental healthcare needs but may also indicate the limits of CPs in terms of their scope of recognising and managing mental illness in community pharmacies.

Evolving and extended roles of CPs in mental healthcare are increasingly being recognised by other healthcare professionals and people seeking mental health support^54^ and offers an opportunity for CPs to be part of an integrated healthcare approach provided that CPs have the necessary MHL encompassing mental health-related knowledge, attitudes and skills. In a UK-based study, CPs were found to have a role in improving mental health and wellbeing for people with mild to moderate depression and anxiety; this was achieved via social prescribing whereby CPs promoted engagement with non-pharmacological pathways related to psychosocial needs in primary care for those experiencing stressors such as isolation, unemployment and unstable accommodation arrangements.^41^

### 4.3 Mental health literacy including stigma

Participants in the current study, despite wanting to provide more personalised and patient-centered care, expressed their concerns about not having enough knowledge and skills to recognise mental illness or have discussions around mental illness with consumers. Indeed, some participants felt more comfortable discussing physical health issues compared to mental health which is in line with previous reports.^30^^,^^38^ Consequently, CPs' may experience discomfort in engaging with consumers with mental health conditions as has been previously reported in the literature.^8^^,^^39^^,^^55^ Additionally, the CPs in the current study described the presence of stigma in the general population that leads to consumers' hesitation in help-seeking but also the presence of stigma in CPs as an additional barrier. Stigma can contribute to health inequities, and stigma towards a number of populations, including those with mental health needs, has been identified in community pharmacy practice with the suggestion of recognising stigma as a problem as the first step followed by methods to address stigma and gaining a better understanding of the social determinants of health.^56^ As pharmacists' exposure to mental health learning is often only during their undergraduate years, there is a need to improve knowledge around the risk factors, causes and recognition of mental illness alongside how to communicate more effectively with those with mental health needs.

### 4.4 Need for training

The provision of ongoing mental health training for pharmacists is reported to be critical in improving the quality of mental healthcare delivery^6^ and represents an important opportunity following completion of undergraduate studies, but currently, may only be self-selected by those who are interested in mental health or those who wished to upskill as opposed to being more widely available to all CPs in NZ. We found several areas of discomfort that could be optimised regarding CPs preparedness in delivering mental healthcare. These include unfamiliarity in providing mental healthcare, anxiety about communicating with or fears of offending those with mental health needs, and mental health impacts on CPs themselves such as stress due to a lack of confidence around psychotropic medications.

With regard to improvements in empathy and communication skills, mental health training including mental health first aid (MHFA), suicide prevention and depression management training in CPs have been associated with positive impacts.^6^ Some participants reported limited exposure to mental health topics in their university curriculum and the overlap of symptoms across a variety of mental health conditions. All participants suggested the need for mental health-related training particularly regarding communication skills and greater understanding of mental health issues which may be during their time at university or once qualified as a pharmacist. Active learning components such as from lived experience accounts, and consumer educators as suggested by the participants in this study, as well as role playing^57^ have been previously identified to improve empathy, self-efficacy, reflection, and insight in health professionals. Ongoing continuing professional development programmes regarding psychotropic medications, communication skills and collaboration with other healthcare professionals would potentially enhance knowledge and confidence among CPs as reported by a study undertaken in the United Arab Emirates.^58^

### 4.5 Community pharmacy as a safe space and meaningful relationships

In addition to MHL, CP services that include individualised, flexible and goal-oriented medication support have been found to enhance consumer outcomes on perception of mental illness, medication adherence and medication beliefs.^59^ Further, mental health help-seekers have reported their relationship with CPs to be critical for mental healthcare as other primary healthcare providers, including their prescribers, have low accessibility.^16^ An Australian study highlighted community pharmacy as a safe space for consumers to discuss their mental health concerns once individuals with mental health needs and their carers developed a trusting relationship with the pharmacists.^47^ In a study investigating patient-pharmacist relationships in general, as opposed to those with mental health needs specifically, the type of pharmacy (independently owned or clinic pharmacy) and consumer expectations of interactions (client or partner) influenced the building of such relationships.^60^

Challenges identified in this study align with the literature on community pharmacy workplace-related factors including the lack of resources and time, and system barriers such as lack of collaboration.^17^^,^^61^, ^62^, ^63^ The lack of time due to the high volume of medication dispensing, inadequate staffing and multiple responsibilities which eventually leads to lack of engagement with consumers was frequently described by participants and this resonates with findings from a Canadian sample where CPs experienced challenges with mental health and addiction care due to inadequate staffing.^16^ According to a recent study, pharmacists in NZ wish to work to top of scope which includes patient-facing roles, but are unable to due to increasing time demands spent on dispensing medications particularly, since the COVID-19 pandemic.^64^ In recent years, NZ has introduced a pharmacy technician role for accuracy checking, with the aim of releasing CPs from a dispensing role to spend more time on patient facing roles and working to top of scope.^65^ Similar roles have been established in the USA (tech-check-tech role) and UK and have proven to be beneficial in terms of enabling pharmacists to spend more time on patient focused activities^66^ and with similar benefits shown in NZ.^67^ However, most participants did not have a skilled technician available in their pharmacies.

In the current study, some pharmacists stated that an increase in chain pharmacies and locums could lead to a lack of a person-centered approach. Moreover, the viewpoint of a business owner can dictate several conditions in the pharmacy setting including workplace staffing levels and the culture within the workplace. In both Australian^51^ and NZ samples,^65^ adequate time and a supportive management structure was necessary to enable clinically focused activities by pharmacists and the opportunity to build relationships.

### 4.6 Community pharmacy and systemic challenges

At a national level, the mental health services specifically funded in NZ community pharmacies are opioid substitution therapy and clozapine monitoring services.^65^ In a study of community pharmacies within NZ, there was much heterogeneity in terms of the unfunded services provided in pharmacies across the country and such services included discussions around weight management, minor ailments and mental health.^45^ In comparison, the Bloom program in Canada^68^ is a CP-led initiative aiming to improve mental health and addictions care in Nova Scotia whereby mental health interventions include access to and information about community mental health and addiction resources, support with navigating the mental health and addictions system, advocacy roles for CPs and with CPs delivering medication management activities. The majority of CPs' activities on the Bloom program were not medication management-related and evaluation of the Bloom program has led to funding from the government for this novel initiative.^69^

The need for better communication and more collaboration with other healthcare professionals was also highlighted. The CPs reported enhanced collaboration and communication in pharmacies co-located with primary care practices, while a lack of collaboration was often due to a lack of time for most healthcare professionals, accessibility of GP or patient records and a lack of understanding of the role of pharmacy services in mental healthcare among other health professionals. These factors are consistent with the literature.^13^^,^^70^^,^^71^ Enhanced communication and collaboration facilitated by technology such as a shared patient platform as suggested in the current study can facilitate better communication between CPs and primary and secondary care services with access to patient notes and collaboration with GPs and psychiatrists being an important consideration.^72^ Future studies should explore the barriers and enablers of such technological approaches along with other methods to enhance integration between various healthcare disciplines.

### 4.7 Implications

In terms of CPs' preparedness to deliver mental health-related services in community pharmacies, respondents felt that NZ's pharmacy curriculum could further focus on improving mental health knowledge and reducing mental health stigma to facilitate CPs in delivering mental healthcare in practice. As pharmacists' exposure to mental health learning is often only during their undergraduate years, there is a need to improve their MHL such that knowledge around the risk factors, causes and recognition of mental illness alongside how to communicate more effectively with those with mental health needs. Though there are several opportunities to address CP preparedness, the proposed solutions would need to be scalable and available to the CP workforce nationally.

While mental healthcare and interventions such as social prescribing are not solely the responsibility of CPs, such an approach would also be important in the NZ-context given that CPs are often an important part of the communities they serve. As with elsewhere, there are social determinants of health and wellbeing in NZ which impact the most socially disadvantaged, particularly Māori.^73^ Te Whare Tapa Wha is a framework that conceptualises health and wellbeing in Māori as a house with four walls that encompass physical, mental, spiritual and family domains, emphasising wider factors in the community, beyond the individual.^74^ Greater involvement of CPs in primary care in the NZ context would potentially allow a more inclusive approach, better integration of care across primary and secondary care settings, and allow the needs of people with mild to moderate mental health conditions to be better addressed.^4^^,^^75^

Most participants in the current study showed enthusiasm and wanted to work at the top of their scope of practice which included clinical interactions with consumers. Participants highlighted the need for more resourcing and funding to actively provide mental healthcare in community settings. With appropriate training and remuneration, CPs can successfully support mental healthcare and work to top of scope of practice.

### 4.8 Strengths and limitations

Although a large number of CPs (n = 85) showed interest in participating in this study by sharing their contact details at the end of our previous quantitative online survey study, there was a low response rate to emails to participate in the current study. This may be due to the long-time interval between this study and the previous study as a result of COVID-19 lockdowns and related impacts. The findings from the interviews should be understood in the setting of community pharmacies just emerging out of the pandemic; if the study were to be conducted again, some of the attitudes and views may potentially vary. It was not possible to assess the demographic information of the 85 potentially interested participants as the survey responses were anonymised and participants were required to fill in a separate survey with their contact details (email and phone number) if they wished to participate in a future qualitative study. This was to ensure that their private details were delinked from their survey responses in the previous quantitative study. Only those potential participants who responded to our invitation to participate in this study were able to provide their demographic details. Thus, it is possible that there was a self-selection bias in this study as participants who are interested in mental health may have been more likely to participate. This is not an uncommon challenge in studies that utilise qualitative interview methodology.^76^ However, a targeted approach of recruiting participants with varying levels of experience and from different pharmacy settings in NZ allowed for potentially representative CPs. Moreover, as much detail about the participants is provided in the current study so that findings can be contextualised and understood. The methodological approach was structured, and all the participants were able to review their transcripts. Preliminary data analysis of early transcripts was conducted by three researchers independently and an iterative process with several discussions to review codes and emerging themes was followed.

The qualitative interview methods facilitated more in-depth exploration of participant views that builds on our previous findings from the survey. Participants may also be CPs who are more open to discussing mental health issues compared to CPs who did not take part in the survey or interviews and therefore may not represent the views of CPs who might be less comfortable with such discussions. Additionally, though there may be CPs who provide extended roles involving mental health support, it is possible that such pharmacists were not captured in our sample. These CPs may have unique perspectives or experiences regarding more specific mental health interventions.

Though we wished to recruit Māori and Pasifika pharmacists to the study, our sample had low levels of representation from pharmacists from these backgrounds and with the sample being composed of largely European and Asian ethnicities. This study is one of the first to evaluate MHL and mental healthcare provided by CPs within NZ, and the findings are of value given the multiethnic make-up of NZ including a unique indigenous Māori population, people from the Pacific islands, Europeans and Asians. The high rates of mental illness particularly in the indigenous population of NZ requires the exploration of potentially novel and community-based approaches to address.

## 5 Conclusion

In this study that explored how MHL may affect the provision and receipt of mental healthcare in community pharmacies as well as the experiences, barriers and facilitators for CPs in providing mental healthcare in community pharmacies in NZ, CPs identified mental illness as complex with several sociocultural determinants and with MHL affecting both help-seeking by consumers and CPs providing mental healthcare. CPs explained that they were more confident and comfortable when addressing the mental health needs of the consumer when they had more professional experience, exposure to mental illness and personal experience of mental health challenges. However, stigma, uncertainty and a lack of knowledge and skills around the understanding, recognition and management of mental illness worsened confidence and comfort for CPs.

CPs highlighted the wish to deliver more consumer facing activities and not only draw on their medicines expertise but there are challenges at the community pharmacy level related to the corporatisation of pharmacies, resourcing and time availability. Factors identified at the system level included a fragmented healthcare system that could potentially affect mental healthcare delivery. CPs wished to work collaboratively with other healthcare professionals to support mental healthcare in an integrated way within primary care and together with secondary/tertiary care. Finally, CPs described ways to enhance preparedness to deliver mental healthcare in community pharmacies including better training opportunities while at university or ongoing professional training opportunities in mental health and more funding for specialist mental healthcare-related services to enable support of those with needs that cannot be met in primary care. Further training that could be useful to improve MHL included enhancing knowledge on the recognition of symptoms of a variety of mental health conditions and how to improve communication and build rapport with consumers. The findings lay the foundation to inform future strategies to promote mental healthcare delivery by CPs.

## CRediT authorship contribution statement

**Frederick Sundram:** Writing – review & editing, Writing – original draft, Supervision, Project administration, Methodology, Investigation, Funding acquisition, Formal analysis, Data curation, Conceptualization. **Amy Hai Yan Chan:** Writing – review & editing, Writing – original draft, Supervision, Project administration, Methodology, Investigation, Funding acquisition, Formal analysis, Data curation, Conceptualization. **Joanne C. Lin:** Writing – original draft, Supervision, Project administration, Methodology, Investigation, Funding acquisition, Formal analysis, Data curation, Conceptualization. **Retina Rimal:** Writing – original draft, Project administration, Investigation, Formal analysis, Data curation. **Timothy F. Chen:** Writing – review & editing, Methodology, Investigation, Funding acquisition. **Jane L. Sheridan:** Writing – review & editing, Writing – original draft, Supervision, Resources, Project administration, Methodology, Investigation, Funding acquisition, Formal analysis, Data curation, Conceptualization.

## Consent for publication

Not applicable.

## Ethics approval and consent to participate

Prior to conducting research activities, the present study was approved by the University of Auckland Human Participants Ethics Committee (UAHPEC Reference ID = 023957). Participants were required to review and sign an informed consent document prior to participating in this study. All participants completed the informed consent form, no participants declined to provide consent.

## Funding

Funding for this study was made available via New Zealand Pharmacy Education and Research Foundation (Grant No: 312).

## Declaration of competing interest

AC has received funding from Oakley Mental Health Foundation and Chorus Ltd. for studies related to mental health, but unrelated to this work. FS has received funding from the Health Research Council and Mindbio Therapeutics, but unrelated to this work. The authors declare that they have no other competing interests.

## Data Availability

The dataset is not publicly available due to information that could compromise the privacy of research participants.

## Appendix A Interview guide

Good morning/good afternoon. Thank you so much for your time today. I am Retina, I am a research assistant for the project on mental health literacy of Community pharmacists (CPs). The purpose of this research is to explore factors that may influence mental health literacy of CPs and its impact on service delivery. There are no right or wrong answers to the questions I am going to put forward. Please feel free to share your views and experiences.

If you feel uncomfortable answering any of the questions, just let me know and we can move on to the next question or if you feel like taking a break or stopping this interview at any time, please feel comfortable to say so. This interview will take up to 60 min of your time and this interview is being recorded for the purpose of transcribing and analysis; however, your information will be deidentified to maintain anonymity.

**Do you have any questions for me before we start?**

• Can you please tell me a little about yourself in terms of years of experience in pharmacy and location of practice?
• We were able to identify your interest in participating in this interview following your completion of the online survey – though it was a while ago, what was your impression of the survey?
• Based on the initial findings of the survey, we found majority of CPs have a good degree of mental health literacy and positive attitudes towards mental health and we would like to explore these and other aspects in more detail during the interview today.

**Understanding of mental illness, attitudes**

• Mental illness can be defined in many different ways, what is your understanding of the term mental illness?
  ○ Probes:
    ▪ How is this different to physical illnesses? Why?
    ▪ What words or emotions come to mind?
• Imagine a person with a mental illness, can you describe them?
  ○ Probes:
    ▪ What do they look like? How would they act?
• Have you recently seen any news on mental illness? How does the media/social media portray mental illness, are they accurate?
• Do you think there is stigma around mental illness? Why do you think that is?
  ○ Probes:
    ▪ What do you think should be done?
    ▪ Role of society/ government/ community pharmacist.
• Personal exposure to mental illness can have an impact on one's MHL, has anyone in your friends/family experienced mental illness
  ○ Probes
    ▪ If yes, do you think this experience has had any effect on your practice?
• If you suspected a friend or family member of yours, had mental health problems, who would you go to for help?
  ○ Probes:
    ▪ Role of society/ medical intervention/ friends and family.
    ▪ Where would you seek help?
    ▪ What would stop you getting help? Why?
    ▪ What would help? Why?

**Expanding on quantitative survey results**

• Our survey respondents suggested they were much more comfortable providing medication advice and managing cardiovascular disease compared to MH needs, why do you think this is?
  ○ Probes:
    ▪ How do you feel?
    ▪ Do you feel confident that you are doing the best you can for them? Why?
• Have you had training on (recognising, treating or preventing?) mental illness?
  ○ Probes:
    ▪ What was the training on and when was it?
    ▪ Is there enough training compared to physical illnesses?
    ▪ If you had the opportunity for training, what would it look like?
    ▪ What do you think of pharmacological and non-pharmacological interventions and diagnostic & counselling skills?
    ▪ Have you accessed PSNZ resources/training for MH conditions? Why? If not, why?
• One of the training scopes that was suggested in the survey included communication with those with MH needs. What are your thoughts?
  ○ Probes
    ▪ How does this interaction differ from your interactions with non-MH service users?
• The front lines of healthcare workers like yourself, can sometimes experience pressure and stress at work, how do you think it should be managed? Would you prefer training on important ways to self-care of pharmacists?

**Exploring community pharmacy service delivery**

• In comparison to depression, Are you comfortable managing or confident of your understanding of other mental illnesses such as schizophrenia or bipolar disorder?
• What have your experiences been like managing those with mental illness presenting to your pharmacy?
  ○ Probes:
    ▪ Any particular cases stand out?
    ▪ What aspects were handled well, what could be improved?
• Do you think managing mental illness is something a community pharmacist should do?
  ○ Probes:
    ▪ If so, what do we need to do to upskill?
    ▪ If not, why not?
    ▪ Do you see any barriers to community pharmacists managing those with mental health needs?
• Do you have a good sense of referral pathways to GPs/specialist mental health services?
  ○ Probe:
    ▪ If you had someone presenting in crisis e.g. acutely suicidal or psychotic, how would you proceed?
• What should community pharmacists, GPs and specialist mental health services manage – is there scope for discussion/collaboration?

**Conclusion**

• Is there anything else you would like to add about mental illness, your own experiences or mental health in general?
• We collected your demographics in the survey, however it was decoupled to maintain anonymity, so we can't link it back to your information. Could we please go over some of the demographics (ethnicity, age, location of your pharmacy and your position in the pharmacy)?
• Thank you for your participation
• Get their postal address for the participant koha^1^
